# Correction: Virome and nrEVEome diversity of *Aedes albopictus* mosquitoes from La Reunion Island and China

**DOI:** 10.1186/s12985-022-01950-8

**Published:** 2022-12-09

**Authors:** Umberto Palatini, Niccolò Alfano, Rebeca Carballar-Lejarazu, Xiao-Guang Chen, Helene Delatte, Mariangela Bonizzoni

**Affiliations:** 1 Department of Biology and Biotechnology, University of Pavia, 27100 Pavia, Italy; 2 Southern Medical University of Guangzhou, Guangzhou, China; 3 CIRAD, La Reunion, France; 4 Present Address: University of California Irvine, Irvine, USA

**Correction : Virology Journal (2022) 19:190** 10.1186/s12985-022-01918-8

Following publication of the original article [1], the authors identified an error in the author name of Rebeca Carballar-Lejarazu.

The incorrect author name is: Rebeca Lejarazu Carballar

The correct author name is: Rebeca Carballar-Lejarazu

The original article [1] has been corrected.
