# Supplementary figures and images for: Virome and nrEVEome diversity of Aedes albopictus mosquitoes from La Reunion Island and China
Source: Virol J. 2022 Nov 18;19:190. doi: 10.1186/s12985-022-01918-8 (PMC9673329; doi:10.1186/s12985-022-01918-8)

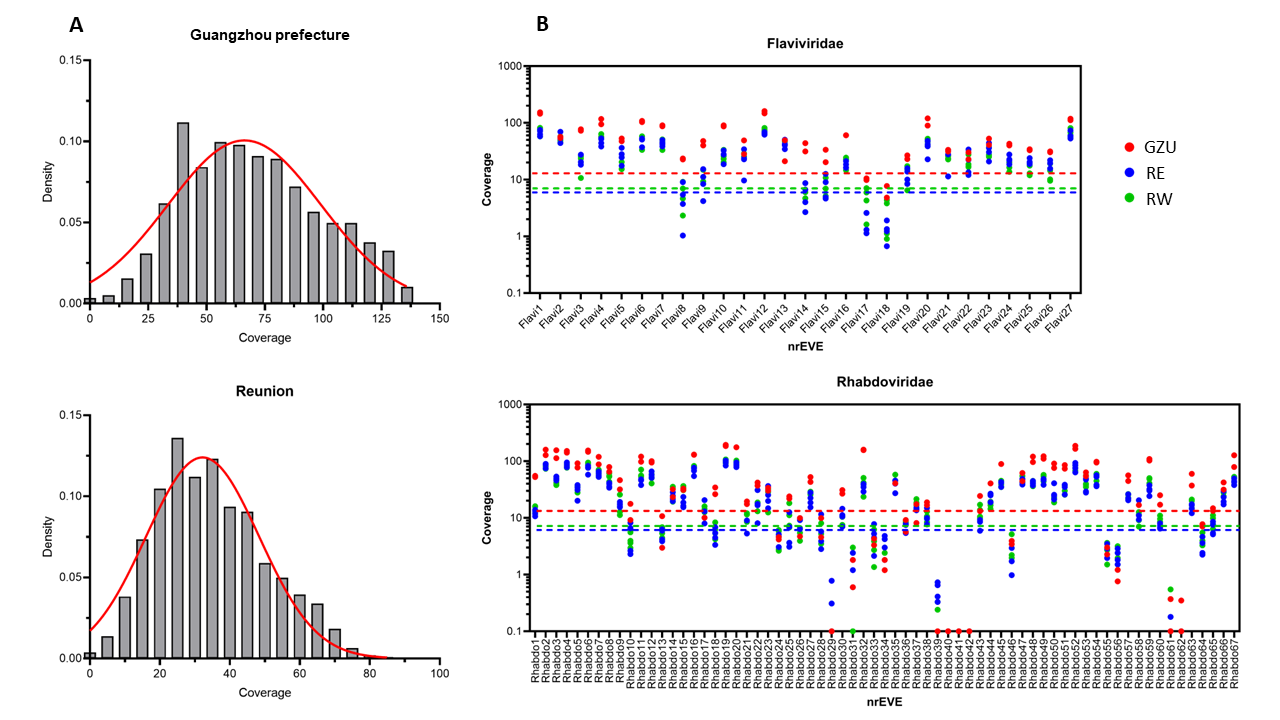

Supplement: Supplementary file 3 — Additional file 3: Fig. S1. Presence or absence of AalbF2 annotated nrEVEs in samples from China and La Reunion. (A) Average distribution of reads coverage for 359 BUSCO genes calculated from the WGS mosquito data from La Reunion island and the Guangzhou prefecture in China described in the study. (B) WGS reads coverage for flaviviridaei- and rhabdoviridae-derived annotated nrEVEs in samples from Guangzhou (GZU) and the Eastern and Western sides of La Reunion Island (RE and RW, respectively). [file 12985_2022_1918_MOESM3_ESM.tif]
